# The Amino Acids Sensing and Utilization in Response to Dietary Aromatic Amino Acid Supplementation in LPS-Induced Inflammation Piglet Model

**DOI:** 10.3389/fnut.2021.819835

**Published:** 2022-01-17

**Authors:** Qing Duanmu, Bie Tan, Jing Wang, Bo Huang, Jianjun Li, Meng Kang, Ke Huang, Qiuchun Deng, Yulong Yin

**Affiliations:** ^1^College of Animal Science and Technology, Hunan Agricultural University, Changsha, China; ^2^Laboratory of Animal Nutritional Physiology and Metabolic Process, Key Laboratory of Agro-Ecological Processes in Subtropical Region, National Engineering Laboratory for Pollution Control and Waste Utilization in Livestock and Poultry Production, Institute of Subtropical Agriculture, Chinese Academy of Sciences, Changsha, China

**Keywords:** aromatic amino acid, amino acids sensing, transporters, sensors, piglets

## Abstract

Dietary supplementation with aromatic amino acids (AAAs) has been demonstrated to alleviate intestinal inflammation induced by lipopolysaccharide (LPS) in the piglets. But the mechanism of AAA sensing and utilization under inflammatory conditions is not well-understood. The study was conducted with 32 weanling piglets using a 2 × 2 factorial arrangement (diet and LPS challenge) in a randomized complete block design. Piglets were fed as basal diet or the basal diet supplemented with 0.16% tryptophan (Trp), 0.41% phenylalanine (Phe), and 0.22% tyrosine (Tyr) for 21 days. The results showed that LPS treatment significantly reduced the concentrations of cholecystokinin (CCK) and total protein but increased leptin concentration, the activities of alanine transaminase, and aspartate aminotransferase in serum. Dietary supplementation with AAAs significantly increased the serum concentrations of CCK, peptide YY (PYY), and total protein but decreased the blood urea nitrogen. LPS challenge reduced the ileal threonine (Thr) digestibility, as well as serum isoleucine (Ile) and Trp concentrations, but increased the serum concentrations of Phe, Thr, histidine (His), alanine (Ala), cysteine (Cys), and serine (Ser) (*P* < 0.05). The serum-free amino acid concentrations of His, lysine (Lys), arginine (Arg), Trp, Tyr, Cys, and the digestibilities of His, Lys, Arg, and Cys were significantly increased by feeding AAA diets (*P* < 0.05). Dietary AAA supplementation significantly increased the serum concentrations of Trp in LPS-challenged piglets (*P* < 0.05). In the jejunal mucosa, LPS increased the contents of Ala and Cys, and the mRNA expressions of solute carrier (SLC) transporters (i.e., SLC7A11, SLC16A10, SLC38A2, and SLC3A2), but decreased Lys and glutamine (Gln) contents, and SLC1A1 mRNA expression (*P* < 0.05). In the ileal mucosa, LPS challenge induced increasing in SLC7A11 and SLC38A2 and decreasing in SLC38A9 and SLC36A1 mRNA expressions, AAAs supplementation significantly decreased mucosal amino acid (AA) concentrations of methionine (Met), Arg, Ala, and Tyr, etc. (*P* < 0.05). And the interaction between AAAs supplementation and LPS challenge significantly altered the expressions of SLC36A1 and SLC38A9 mRNA (*P* < 0.05). Together, these findings indicated that AAAs supplementation promoted the AAs absorption and utilization in the small intestine of piglets and increased the mRNA expressions of SLC transports to meet the high demands for specific AAs in response to inflammation and immune response.

## Introduction

During the immunological stress, amino acids (AAs) are redistributed away from protein production toward tissues involved in inflammation and immune response (1–3). The metabolism reprogramming in the immune process could affect the animal's ability to sense and demand AAs because AAs are used as a substrate for the synthesis of inflammatory proteins and immunoglobulins (4). Therefore, the transportation and metabolism of AA are important for immune cells. Immunological stress and inflammation will lead to the increase of basal metabolic rate, which directly leads to metabolic changes (5). The increased synthesis of immune system metabolites such as acute phase proteins, immunoglobulin, and glutathione is accompanied by the increased demand for specific AA (6). For example, the dietary tyrosine (Tyr), phenylalanine (Phe), and tryptophan (Trp) requirements are increased to support the immune response under inflammation conditions in pigs (7). Circulating aromatic AAs (AAAs) as the crucial mediators in the communication between gut and brain participates in immune regulation (8).

The absorptions of dietary AAs by the small intestine play critical roles on extraintestinal tissues and the serum AA profiles are correlated with the mRNA expression levels for key AA transporters in the small intestine (9). Some AA transporters are transceptors with both transporting and sensing functions, which trigger the downstream signal transduction pathway such as the Target of Rapamycin Complex 1 (mTORC1) pathway and general control non-derepressible kinase pathway (10–13). The previous study has demonstrated that dietary supplementation with AAAs activated the Ca^2+^-sensing receptor (CaSR) signaling pathway and alleviated intestinal inflammation induced by LPS in piglets (14). CaSR is expressed in the enterocytes cells throughout the intestine and responds to a broad range of AAs, especially aromatic compounds (15). CaSR couples to the phosphatidylinositol pathway and has been linked to AAAs stimulation of CCK release and intracellular Ca^2+^ mobilization (16). It has been extensively documented that CaSR coordinated food digestion and nutrient absorption, promoted cell proliferation and differentiation, regulated energy metabolism and immune response, stimulated hormone secretion, mitigated secretory diarrhea, and enhanced intestinal barrier function (17–19). This would appear to provide a molecular explanation for AA absorption and utilization to support the inflammatory response in the intestine. The signaling pathways downstream of CaSR, phospholipase Cβ2, and NF-κB have been confirmed to be involved in the regulation of AAAs on intestinal inflammation (14). But it is not clear whether the CaSR activation is accompanied by the change of availability of AAs.

Therefore, the present study is conducted to investigate the effects of dietary supplementation with AAAs on the ileal apparent digestibility of AAs, serum and mucosal AAs profiles as well as AA transporters in the small intestine of LPS-challenged piglets.

## Materials and Methods

The animal trial was approved by the Institutional Animal Care and Use Committee of the Institute of Subtropical Agriculture, Chinese Academy of Sciences (2013020).

### Animal Experiment Design

The animal experimental design was based on the same experimental protocol that has been presented by Liu et al. (14). Briefly, a total of 32 cross-bred (Duroc × Landrace) weanling gilts and barrows (6.66 ± 0.31 kg body weight) were randomly assigned into four treatments (eight piglets /treatment) using a 2 × 2 factorial arrangement. The main factors were dietary treatment (piglets were fed the basal diet or the 0.16% Trp, 0.41% Phe, and 0.22% Tyr supplemented diet) and LPS challenge (piglets were challenged with LPS or treated with sterile saline). The diets preparation, feeding, and management of piglets were the same as the description in the previous study (14).

On the morning of day 21 after the initiation of the treatment, the piglets were intraperitoneally injected with either 100 μg/kg BW LPS (*Escherichia coli* strain O5:B55) or the same volume of 0.9% sterilized saline, respectively. Blood samples were collected from the jugular vein at 4 h after injection and serum samples were obtained by centrifugation at 2,000 g for 15 min and then stored at −80°C until further analysis. Jejunal and ileal mucosa were collected and immediately snap-frozen in liquid nitrogen and stored at −80°C for the analysis of free AA profiles and gene expression. In addition, digesta samples were collected from terminal ileum for the AAs digestibility analysis.

### Analysis of Serum Metabolites and Hormones

Serum biochemical parameters, including total protein (TP), albumin (ALB), alkaline phosphatase (ALP), alanine transaminase (ALT), aspartate aminotransferase (AST), blood urea nitrogen (BUN), glucose (GLU), and lactic dehydrogenase (LDH), were measured using Biochemical Analytical Instrument (Beckman CX4) and commercial kits (Sino-German Beijing Leadman Biotech Ltd, Beijing, China).

Serum samples were treated with sulfosalicylic acid, centrifuged, and filtered through a 0.45 μm filtration membrane. Then the amino acid concentrations were determined using an automatic amino acid analyzer (Model L-8900, Hitachi Ltd. Tokyo, Japan).

The serum concentrations of cholecystokinin (CCK), peptide YY (PYY), ghrelin, glucagon, and leptin were determined using the corresponding pig ELISA Kit (CUSABIO, Wuhan, China) in accordance with the manufacturer's instructions.

### Determination of Free Amino Acids in the Intestinal Mucosa

About 0.5 g of jejunal and ileum mucosal tissues were weighed, and 5 ml of 0.1 M HCl homogenate was added, centrifuged for 10 min at 5,000 g. We took 0.5 ml of the supernatant, mixed it with the same volume of 8% sulfosalicylic acid, and left it resting at 4°C overnight. Centrifugation was performed at 12,000 g for 10 min. Then the supernatant was absorbed and centrifuged again at 12,000 g for 10 min, and then through filter membranes. Then the AA content was determined by a high-performance liquid chromatography (Agilent 1200, Agilent Technologies, USA). The test conditions were as following: wavelength: 254 nm; flow rate: 1 ml/min; column temperature: elution at 40°C; Acetonitrile: 0.02 mol/L; Ammonium formate = 30:70 (V:V).

### Digestibility of Amino Acid Analysis

Feed samples and terminal ileal digesta samples (0.5 g) were accurately weighed and put into an ampere tube, 10 ml of 6 M hydrochloric acid was added. The tube was sealed with an alcohol torch, hydrolyzed at 110 ± 2°C for 24 h, and then transferred to a 100 ml volumetric flask after cooling. Took a constant volume of 1–25 ml from the above solution. Then filtered into the injection flask with a 0.22 μm membrane. The AAs content was determined by high-performance liquid chromatography (Agilent 1200, Agilent Technologies, USA).

The feed samples and ileal digesta after freeze-drying were weighed in parallel samples for analysis and determination. The AA profiles were detected by high-performance liquid chromatography (Agilent 1200, Agilent Technologies, USA). Lysine and threonine were detected after hydrolyzing with 6 mol/L HCl at 105°C for 24 h. Methionine was analyzed as methionine sulfone after cold performic acid oxidation overnight before hydrolysis. Tryptophan was determined after hydrolyzing with 4 mol/L LiOH at 110°C for 20 h. The apparent ileal digestibility (AID) of AAs was calculated using the following formula:


$$\begin{array}{l} \text{AID of diet component }=\;[(\text{Diet component}/\text{Chromium})\text{d}\\ -(\text{Diet component}/\text{Chromium})\text{ i}]\\ {}*1/(\text{Dietcomponent}/\text{Chromium})\text{ d}. \end{array}$$

Where (Diet component/Chromium) d = ratio of diet component to Chromium in the diet and (Diet component/ Chromium) i = ratio of diet component to Chromium in the ileal digesta (20, 21).

### Real-Time Quantitative RT-PCR

Total RNA was isolated from the liquid nitrogen-pulverized jejunal, and ileal mucosa samples, and cDNA were synthesized as previously described (9). The mRNA abundance of AA transporters including SLC1A1, SLC7A11, SLC1A5, SLC6A19, SLC6A20, SLC16A10, SLC36A1, SLC38A2, SLC38A9, SLC3A1, SLC6A14, SLC7A1, SLC7A2, SLC7A7, SLC7A9, and SLC3A2 were analyzed using quantitative real-time polymerase chain reaction analysis. The primer sequences for the tested genes are shown the previous study (9). Data are expressed as the relative values to those for piglets of the basal diet with saline injection treatment.

### Statistical Analysis

All data were analyzed by ANOVA using the general linear model procedures of SPSS for a 2 × 2 factorial design (SPSS Inc., Chicago, IL, USA, 2001). The statistical model included the effects of challenge (saline or LPS), diet (basal or AAAs), and their interactions. When there was significant interaction. The differences among treatments were evaluated using the Duncan test. *P* < 0.05 was considered significant.

## Results

### Serum Concentrations of Gastrointestinal Hormones

Compared to the saline injected piglets, LPS administration remarkably decreased the serum CCK and increased leptin concentration. However, the supplementation of AAAs in the diet markedly increased the concentrations of CCK and PYY (*P* < 0.05). LPS challenge × diet had an interactive effect on serum leptin concentration (*P* < 0.05). There were no significant differences in serum concentrations of ghrelin and glucagon among all treatments (*P* > 0.05) (Table 1).

**Table 1:** Effects of dietary supplementation with AAA on serum concentrations of gastrointestinal hormones in piglets (pg/ml).

**Item**	**Basal diet**		**AAA diet**		**SEM**	***P*** **-value**		
	**Saline**	**LPS**	**Saline**	**LPS**		**Diet**	**LPS**	**Diet × LPS**
Cholecystokinin	234.23	186.89	286.76	265.35	15.918	<0.001	0.041	0.425
Peptide YY	305.58	278.95	387.45	328.64	27.223	0.024	0.134	0.565
Ghrelin	457.52	476.50	487.82	457.98	33.868	0.866	0.876	0.485
Glucagon	187.85	168.98	205.65	201.36	15.068	0.116	0.461	0.641
Leptin	221.06^b^	299.64^a^	275.64^a^	254.65^ab^	13.650	0.734	0.049	0.001

*AAA diet, basal diet supplemented with 0.16% tryptophan + 0.41% phenylalanine + 0.22% tyrosine*.

a, b *Within a row, means sharing different superscript letters differ significantly (p < 0.05)*.

### Serum Biochemical Parameters

As shown in Table 2, LPS treatment markedly increased the activity of alanine transaminase and aspartate aminotransferase but decreased the serum concentration of total protein (*P* < 0.05). Dietary AAAs supplementation increased the serum concentration of total protein and decreased the concentration of blood urea nitrogen (*P* < 0.05). There was no significant change of other determined serum biochemical parameters in response to LPS or AAA treatment (*P* > 0.05).

**Table 2:** Effects of dietary supplementation with AAA on serum biochemical parameters (*P* < 0.05).

**Item**	**Basal diet**		**AAA diet**		**SEM**	***P*** **-value**		
	**Saline**	**LPS**	**Saline**	**LPS**		**Diet**	**LPS**	**Diet × LPS**
Total protein (g/L)	74.12	69.32	83.87	76.56	1.843	<0.001	0.004	0.520
Albumin (g/L)	36.45	39.46	38.58	37.48	1.536	0.963	0.558	0.214
Alkaline phosphatase (U/L)	198.45	189.25	215.69	208.96	16.762	0.290	0.645	0.943
Alanine transaminase (U/L)	48.95	64.52	50.42	58.35	3.381	0.505	0.002	0.282
Aspartate aminotransferase (U/L)	53.64	72.54	52.98	60.54	5.004	0.231	0.016	0.282
Blood urea nitrogen (mmol/L)	4.23	4.56	3.68	4.05	0.240	0.043	0.168	0.923
Glucose (mmol/L)	4.87	4.77	4.54	4.39	0.319	0.303	0.710	0.942
Lactic dehydrogenase (U/L)	784.56	851.54	778.65	825.45	30.108	0.621	0.086	0.755

*AAA diet, basal diet supplemented with 0.16% tryptophan + 0.41% phenylalanine + 0.22% tyrosine*.

### Serum Amino Acids Profiles

The results of serum AAs profiles were shown in Table 3. LPS treatment induced the increases in serum concentrations such as His, Phe, Thr, Ala, Cys, and Ser, also including total non-essential amino acids (NEAA), but the decreases of serum Ile and Trp concentrations (*P* < 0.05). Dietary supplementation of the AAAs improved the serum concentrations of His, Lys, Arg, Trp, Tyr, and Cys (*P* < 0.05). An interaction of LPS challenge × diet was observed for Trp content (*P* < 0.05).

**Table 3:** Effects of dietary supplementation with AAA on serum concentration of amino acids in piglets (μmol/L).

**Item**	**Basal diet**		**AAA diet**		**SEM**	***P*** **-value**		
	**Saline**	**LPS**	**Saline**	**LPS**		**Diet**	**LPS**	**Diet × LPS**
His	41.11	53.53	41.91	65.89	1.464	0.034	<0.001	0.060
Ile	73.42	58.20	71.97	55.51	2.912	0.726	0.012	0.916
Leu	104.81	89.05	89.97	87.72	4.722	0.400	0.350	0.481
Lys	142.67	128.09	155.06	157.64	4.771	0.038	0.536	0.378
Met	17.09	18.64	21.55	19.98	0.950	0.140	0.994	0.419
Phe	56.87	67.77	64.67	73.19	2.238	0.153	0.040	0.793
Arg	45.85	46.40	56.06	48.26	1.431	0.046	0.217	0.158
Thr	24.55	28.66	22.10	35.14	1.705	0.560	0.019	0.203
Trp	15.45^b^	14.18^b^	26.41^a^	15.30^b^	1.123	0.013	0.011	0.038
Val	79.59	76.49	71.65	81.23	2.468	0.749	0.518	0.212
EAA	601.42	581.01	621.36	639.85	17.224	0.264	0.978	0.578
Ala	545.30	770.11	494.02	930.11	27.690	0.336	<0.001	0.068
Asn	6.33	5.99	6.18	6.41	0.165	0.688	0.868	0.397
Asp	5.14	6.68	4.86	4.40	0.567	0.270	0.639	0.387
Cys	1.21	1.96	1.38	2.66	0.097	0.034	<0.001	0.192
Gln	338.58	362.71	322.47	364.73	9.618	0.717	0.097	0.642
Glu	91.14	105.17	87.97	88.88	7.449	0.520	0.621	0.664
Gly	542.11	538.29	500.34	593.69	21.636	0.876	0.311	0.273
Pro	167.77	180.06	161.81	200.81	8.197	0.656	0.131	0.423
Ser	74.69	83.19	71.81	96.37	2.222	0.258	0.001	0.084
Tyr	37.55	42.77	64.33	58.03	3.457	0.006	0.938	0.413
NEAA	1809.81	2096.94	1715.18	2346.08	54.062	0.482	<0.001	0.125

*AAA diet, basal diet supplemented with 0.16% tryptophan + 0.41% phenylalanine + 0.22% tyrosine*.

a, b *Within a row, means sharing different superscript letters differ significantly (p < 0.05)*.

*His, histidine; Ile, isoleucine; Leu, leucine; Lys, lysine; Met, methionine; Phe, phenylalanine; Arg, arginine; Thr, threonine; Trp, tryptophan; Val, valine; EAA, essential amino acid; Ala, alanine; Asn, asparagine; Asp, aspartic acid; Cys, cysteine; Gln, glutamine; Glu, glutamic acid; Gly, glycine; Pro, proline; Ser, serine; Tyr, tyrosine; NEAA, non-essential amino acid*.

### The Jejunal and Ileal Mucosal Amino Acid Profiles and Apparent Ileal Digestibility of Amino Acid

In the jejunal mucosa, LPS significantly increased the contents of Ala and Cys but decreased the Lys and Gln contents (*P* < 0.05). Dietary supplementation with AAA had interactive effects with LPS (Diet × LPS, *P* < 0.05) on the Gly and Ser contents (Table 4).

**Table 4:** Effects of dietary supplementation with AAA on the concentration of amino acid content in the jejunal mucosa (μg/g).

**Item**	**Basal diet**		**AAA diet**		**SEM**	***P*** **-value**		
	**Saline**	**LPS**	**Saline**	**LPS**		**Diet**	**LPS**	**Diet × LPS**
His	83.02	72.00	77.34	79.21	1.910	0.842	0.242	0.103
Ile	74.95	64.83	73.59	74.09	2.255	0.389	0.296	0.249
Leu	176.99	160.13	170.39	177.97	4.341	0.523	0.597	0.171
Lys	78.67	64.14	71.15	55.19	3.097	0.195	0.021	0.909
Met	88.19	79.37	83.92	84.82	2.212	0.895	0.379	0.282
Phe	111.15	103.26	108.41	114.15	2.975	0.499	0.858	0.262
Arg	66.06	55.30	57.77	52.59	2.577	0.295	0.134	0.593
Thr	134.90	122.83	126.40	136.15	3.542	0.736	0.871	0.135
Trp	30.17	27.52	31.12	31.13	0.764	0.148	0.395	0.392
Val	125.70	116.41	122.23	130.71	3.346	0.426	0.952	0.195
EAA	969.81	865.81	922.32	936.01	22.276	0.801	0.320	0.198
Ala	353.84	374.15	322.71	411.29	9.109	0.870	0.006	0.072
Asn	18.45	16.25	17.45	17.86	0.433	0.730	0.310	0.143
Asp	225.09	220.01	234.56	230.17	6.681	0.469	0.726	0.980
Cys	3.69	5.40	3.75	4.55	0.265	0.457	0.025	0.397
Gln	234.66	199.89	229.17	206.98	6.636	0.952	0.041	0.639
Glu	1030.23	1011.80	1000.10	1061.88	24.635	0.841	0.664	0.423
Gly	510.27^a^	477.69^ab^	430.61^b^	496.87^a^	10.373	0.156	0.424	0.025
Pro	215.78	197.55	204.71	217.54	5.869	0.707	0.820	0.197
Ser	243.80^a^	210.56^b^	218.77^ab^	229.48^ab^	4.920	0.758	0.263	0.034
Tyr	120.98	112.38	118.82	125.90	3.088	0.366	0.903	0.215
NEAA	2956.79	2825.69	2780.66	3002.53	55.436	0.997	0.686	0.123

*AAA diet, basal diet supplemented with 0.16% tryptophan + 0.41% phenylalanine + 0.22% tyrosine*.

a, b *Within a row, means sharing different superscript letters differ significantly (p < 0.05)*.

*His, histidine; Ile, isoleucine; Leu, leucine; Lys, lysine; Met, methionine; Phe, phenylalanine; Arg, arginine; Thr, threonine; Trp, tryptophan; Val, valine; EAA, essential amino acid; Ala, alanine; Asn, asparagine; Asp, aspartic acid; Cys, cysteine; Gln, glutamine; Glu, glutamic acid; Gly, glycine; Pro, proline; Ser, serine; Tyr, tyrosine; NEAA, non-essential amino acid*.

In the ileal mucosa, LPS injection had no effects on all determined AAs (*P* > 0.05). Dietary supplementation with AAA decreased the contents of Ile, Leu, Met, Phe, Thr, Val, Ala, Asn, Gly, Pro, Ser, and Tyr, as well as the total NEAA (*P* < 0.05). There was no interaction of LPS challenge × diet on all determined AAs contents (*P* > 0.05) (Table 5).

**Table 5:** Effects of dietary supplementation with AAA on the concentration of amino acid content in the ileal mucosa (μg/g).

**Item**	**Basal diet**		**AAA diet**		**SEM**	***P*** **-value**		
	**Saline**	**LPS**	**Saline**	**LPS**		**Diet**	**LPS**	**Diet × LPS**
His	97.12	102.10	84.15	87.81	3.534	0.064	0.545	0.926
Ile	91.30	82.23	73.00	65.79	3.277	0.013	0.225	0.887
Leu	206.45	191.90	171.25	162.09	6.771	0.023	0.389	0.843
Lys	100.14	86.03	79.93	86.77	5.607	0.393	0.748	0.358
Met	99.65	96.74	85.29	81.92	2.897	0.018	0.592	0.969
Phe	116.65	112.93	100.31	96.28	3.762	0.037	0.610	0.984
Arg	81.12	71.18	61.95	62.22	3.526	0.056	0.498	0.475
Thr	162.71	160.30	135.51	137.04	5.654	0.034	0.969	0.863
Trp	31.97	31.34	29.04	28.15	1.190	0.209	0.753	0.955
Val	164.05	146.18	125.69	120.73	5.462	0.007	0.305	0.559
EAA	788.95	1080.92	946.11	928.82	59.833	0.983	0.261	0.207
Ala	440.10	439.94	314.94	357.64	12.670	<0.001	0.408	0.405
Asn	18.55	16.01	14.48	14.43	0.531	0.013	0.234	0.249
Asp	243.43	235.79	227.48	218.17	8.421	0.328	0.619	0.961
Cys	1.71	1.39	1.50	1.48	0.095	0.743	0.369	0.423
Gln	143.77	189.18	177.70	166.22	12.965	0.834	0.518	0.282
Glu	1228.95	1255.79	1211.44	1193.44	32.344	0.542	0.946	0.731
Gly	648.80	591.85	493.94	535.14	14.173	0.001	0.783	0.094
Pro	284.51	256.71	217.11	205.94	9.596	0.005	0.319	0.668
Ser	328.64	270.07	226.76	223.76	8.696	<0.001	0.088	0.121
Tyr	124.46	125.24	110.75	107.91	3.702	0.045	0.891	0.809
NEAA	3462.92	3381.98	2996.09	3024.15	83.059	0.019	0.875	0.745

*AAA diet, basal diet supplemented with 0.16% tryptophan + 0.41% phenylalanine + 0.22% tyrosine*.

*His, histidine; Ile, isoleucine; Leu, leucine; Lys, lysine; Met, methionine; Phe, phenylalanine; Arg, arginine; Thr, threonine; Trp, tryptophan; Val, valine; EAA, essential amino acid; Ala, alanine; Asn, asparagine; Asp, aspartic acid; Cys, cysteine; Gln, glutamine; Glu, glutamic acid; Gly, glycine; Pro, proline; Ser, serine; Tyr, tyrosine; NEAA, non-essential amino acid*.

The apparent ileal digestibility of AAs was shown in Table 6, LPS significantly increased the digestibility of His but decreased the digestibility of Thr. Dietary AAA supplementation enhanced the digestibility of His, Lys, Arg, and Cys (*P* < 0.05). There was no interaction of LPS challenge × diet on the apparent ileal digestibility of AAs (*P* > 0.05).

**Table 6:** Effects of dietary supplementation with AAA on apparent ileal digestibility of amino acid in piglets.

**Item**	**Basal diet**		**AAA diet**		**SEM**	***P*** **-value**		
	**Saline**	**LPS**	**Saline**	**LPS**		**Diet**	**LPS**	**Diet × LPS**
**Essential AA (%)**
His	83.32	85.45	85.46	88.98	1.298	0.043	0.044	0.608
Ile	69.45	70.59	72.59	71.54	1.168	0.098	0.970	0.366
Leu	67.62	66.78	67.97	70.89	1.321	0.116	0.455	0.182
Lys	83.56	82.50	86.54	85.57	1.276	0.032	0.454	0.973
Met	86.89	85.48	87.64	86.49	1.579	0.594	0.440	0.936
Phe	88.96	87.46	91.54	89.45	1.254	0.099	0.190	0.827
Arg	78.79	79.54	83.74	80.38	1.316	0.047	0.356	0.150
Thr	73.58	68.45	74.87	72.87	1.393	0.060	0.021	0.291
Trp	75.45	73.59	76.89	77.74	1.367	0.061	0.726	0.351
Val	72.55	70.57	74.86	73.46	1.259	0.055	0.203	0.830
**Non-essential AA (%)**
Ala	65.43	64.78	68.99	66.46	1.910	0.303	0.710	0.942
Asp	79.46	80.58	81.78	82.69	1.502	0.162	0.518	0.945
Cys	76.84	74.86	77.55	79.54	1.275	0.049	0.995	0.140
Gln	80.79	78.49	81.05	81.57	1.176	0.183	0.474	0.260
Gly	71.58	72.41	72.68	73.96	1.338	0.363	0.468	0.876
Pro	76.89	74.15	78.85	77.68	1.667	0.120	0.262	0.649
Ser	77.95	75.87	79.87	78.68	1.468	0.138	0.300	0.774
Tyr	71.58	69.87	73.58	72.69	1.122	0.056	0.289	0.738

*AAA diet, basal diet supplemented with 0.16% tryptophan + 0.41% phenylalanine + 0.22% tyrosine*.

*His, histidine; Ile, isoleucine; Leu, leucine; Lys, lysine; Met, methionine; Phe, phenylalanine; Arg, arginine; Thr, threonine; Trp, tryptophan; Val, valine; Ala, alanine; Asp, aspartic acid; Cys, cysteine; Gln, glutamine; Gly, glycine; Pro, proline; Ser, serine; Tyr, tyrosine*.

### The MRNA Expression Level of Amino Acids Transporters in the Jejunal and Ileal Mucosa

In the jejunum, the relative mRNA expressions of SLC7A11, SLC16A10, SLC38A2, SLC6A14, SLC3A2, and SLC7A2 were markedly increased but the expression of SLC1A1 was decreased by LPS challenge (*P* < 0.05). AAAs supplementation only decreased the expression of SLC6A19 mRNA (*P* < 0.05). The interaction between AAAs supplementation and LPS challenge notably affected the expression of SLC1A1, SLC36A1, SLC3A1, SLC7A2, and SLC7A9 mRNA (*P* < 0.05) (Table 7).

**Table 7:** Effects of dietary supplementation with AAA on the mRNA expression level of amino acids transporters in the jejunum and ileum of piglets (*P* < 0.05).

**Item**	**Basal diet**		**AAA diet**		**SEM**	***P*** **-value**		
	**Saline**	**LPS**	**Saline**	**LPS**		**Diet**	**LPS**	**Diet × LPS**
**Jejunum**
SLC1A1	1.00^b^	0.89^b^	1.71^a^	0.76^b^	0.088	0.113	0.006	0.024
SLC7A11	1.00	6.61	1.03	4.71	0.438	0.296	<0.001	0.281
SLC1A5	1.00	0.76	0.67	1.35	0.113	0.348	0.568	0.054
SLC6A19	1.00	0.79	0.62	0.46	0.076	0.028	0.241	0.871
SLC6A20	1.00	0.92	0.90	0.95	0.074	0.822	0.933	0.659
SLC16A10	1.00	1.19	0.65	1.79	0.121	0.601	0.011	0.060
SLC36A1	1.00^ab^	0.87^ab^	0.44^b^	1.33^a^	0.105	0.825	0.084	0.022
SLC38A2	1.00	1.91	0.88	1.60	0.162	0.517	0.018	0.774
SLC38A9	1.00	0.79	1.16	1.14	0.090	0.168	0.528	0.596
SLC3A1	1.00^ab^	0.61^b^	0.67^b^	1.53^a^	0.120	0.234	0.338	0.016
SLC6A14	1.00	1.25	0.73	2.25	0.157	0.254	0.009	0.054
SLC7A1	1.00	1.55	0.81	1.72	0.144	0.973	0.017	0.529
SLC7A2	1.00^b^	2.27^ab^	0.58^b^	5.74^a^	0.456	0.106	0.002	0.042
SLC7A7	1.00	1.03	0.60	1.08	0.090	0.331	0.168	0.228
SLC7A9	1.00^a^	0.41^c^	0.46^bc^	0.89^ab^	0.059	0.798	0.530	<0.001
SLC3A2	1.00	1.65	1.24	1.87	0.150	0.445	0.043	0.969
**Ileum**
SLC1A1	1.00	0.49	1.17	1.30	0.118	0.051	0.427	0.1911
SLC7A11	1.00	2.42	0.49	3.20	0.266	0.798	0.001	0.237
SLC1A5	1.00	1.23	1.02	2.26	0.256	0.317	0.164	0.334
SLC6A19	1.00	0.70	0.76	0.75	0.080	0.566	0.335	0.374
SLC6A20	1.00	0.60	0.94	1.11	0.101	0.273	0.557	0.171
SLC16A10	1.00	1.44	2.10	3.26	0.243	0.007	0.116	0.466
SLC36A1	1.00^a^	0.34^b^	0.39^a^	0.41^b^	0.067	0.057	0.026	0.018
SLC38A2	1.00	1.83	1.46	3.14	0.224	0.060	0.010	0.350
SLC38A9	1.00^b^	0.98^b^	3.19^a^	1.63^b^	0.158	<0.001	0.020	0.024
SLC3A1	1.00	0.59	1.10	1.30	0.187	0.293	0.780	0.423
SLC6A14	1.00	0.87	1.01	1.79	0.184	0.219	0.389	0.231
SLC7A1	1.00	0.73	0.40	0.99	0.113	0.457	0.491	0.075
SLC7A2	1.00	0.92	0.66	1.16	0.098	0.814	0.296	0.150
SLC7A7	1.00	1.33	1.22	1.66	0.120	0.266	0.123	0.810
SLC7A9	1.00	0.42	1.11	1.29	0.208	0.251	0.629	0.375
SLC3A2	1.00	0.95	0.85	0.97	0.112	0.783	0.871	0.699

*AAA diet, basal diet supplemented with 0.16% tryptophan + 0.41% phenylalanine + 0.22% tyrosine*.

*The mRNA expression levels normalized basal diet LPS treatment group, AAA diet saline, and LPS treatment group by basal diet saline treatment group*.

a, b, c *Within a row, means sharing different superscript letters differ significantly (p < 0.05)*.

In the ileum, the AAAs supplementation had significantly up-regulated the mRNA expressions of SLC16A10 and SLC38A9 (*P* < 0.05). The mRNA expression of SLC38A2 and SLC7A11 were increased, but the expression of SLC36A1 was decreased by the LPS challenge (*P* < 0.05). The interaction between AAAs supplementation and the LPS challenge significantly altered the expression of SLC36A1 and SLC38A9 (*P* < 0.05) (Table 7).

## Discussion

The immune system stimulation alters the animals' physiology and metabolism via a complex system involving innate and adaptive immune response, several cytokines and acute-phase proteins, as well as the central nervous system (22). During the period of immune system stimulation, especially the metabolism and demand of Glu, Arg, Trp, Thr, and sulfur-containing AAs undergo certain changes (23). The present study showed the changes of AAs metabolism in piglets in response to LPS change including the AAs profiles in serum and intestinal mucosa, as well as apparent ileal digestibility of AA, etc. However, dietary supplementation with AAAs showed to improve the AAs sensing and utilization under inflammatory conditions.

Firstly, LPS significantly reduced the amount of CCK and increased the concentrations of leptin. But supplementation with AAAs counteracted the negative effect of LPS and stimulated CCK secretion. It has been well-documented that AAs stimulate cholecystokinin release through the Ca^2+^-sensing receptor (14). In non-calcified tissues, CaSR affected gastrointestinal nutrient sensing and intestinal endocrine hormone secretion (24). Additionally, it is allosterically sensed and associated with AAAs (15). As a metabolic substrate for Clostridium sporogenes, AAAs could be metabolized into 12 compounds, 9 of which could accumulate in the serum and affect systemic immunity (25). Trp and Phe were the most potent CaSR activators in ${\text{Ca}}_{\text{i}}^{2+}$ mobilization assays (26). The supplementation of AAAs alleviated intestinal inflammation mediated by the CaSR signaling pathway (14). CaSR mediated the secretion of CCK induced by AAAs in the native intestinal I cell. And L-Phe stimulated CCK secretion enhancement in the presence of extracellular calcium levels (27). L-Phe increased serum glucagon and PYY levels but reduced the ghrelin levels in plasma (28).

Secondly, the addition of AAAs not only increased the total protein levels but also significantly reduced the blood urea nitrogen content, which partly indicated the decrease of N excretion and increase of AAs utilization in piglets. The increased N excretion, which occurs during the immune response, is a reflection of a relative imbalance in the profile of AAs released from peripheral tissues (29). Numerous compelling investigations have indicated that a metabolic alteration will occur in intestinal inflammation, resulting in the change of serum profile of AAs (30–33). LPS challenge reduced the ileal Thr digestibility, as well as serum total protein, Ile, and Trp concentrations, but increased the serum concentrations of Phe, Thr, His, Ala, Cys, and Ser in the present study. Reduced total protein means that AA is redistributes inflammation and immunity, not protein synthesis (5, 34). Therefore, it requires the increased provision of particular AAs from the diet in order to spare body protein stores (29). Some dispensable AAs become limiting because their *de novo* synthesis could be impaired. Several strands of evidence suggest that sulfur AAs, and AAs that are metabolically related to them, may be required in increased amounts (35). The demand for Cys increases under immune system stimulation and is used for the synthesis of glutathione (36) and acute phase proteins (22). AAAs demands are also increased to support the immune response under inflammation conditions in pigs (12). Therefore, dietary supplementation with AAAs showed the anti-inflammatory effects in LPS-challenged piglets (10), which could be explained by the increase in AID of His, Lys, Arg, and Cys, as well as serum concentrations of His, Arg, Trp, and Cys. The increased levels of AAs in serum may be due to the increase in hepatic catabolism and AA requirements for utilization. Interestingly, the levels of 12 kinds of AA were reduced in the jejunal mucosa, where after only the level of Trp was elevated significantly in serum. This is because the capture, absorption, transformation, and metabolism of intestinal epithelial cells and liver metabolism were jointly responsible for regulating the amount of AAs in peripheral blood (37, 38). It was also found in rat liver that His, Phe, Leu, Tyr, Gln, Pro, Trp, and Met inhibit intracellular proteolysis (39). And AA catabolism by the mucosal cells was quantitatively greater than AA incorporation into mucosal protein (40). The catabolism of AAs in the small intestine plays an important role in regulating the availability of dietary AAs to extraintestinal tissues (41).

It is well-known that AAs are absorbed through AA transporters, which may act as an initiator of nutritional signaling. Signaling pathways are intrinsically linked to amino acid transporter activity as well as to intracellular AAs metabolism (42). The SLC family mediates the transport of AAs on the plasma membrane (43). Both A2 and A9 of the SLC38 family are involved in the mTOR pathway. SLC38A9, which is a lysosomal Arg sensor machinery in the mTORC1 pathway (44). The Arg activates mTORC1 through the SLC38A9 sensor and binds it to other essential AAs in lysosomes as a lysosomal messenger, including Phe, Leu, Ile, Trp, Tyr, Val, Pro, Ser, and Met (45). The expression of SLC38A9 in the ileum and the content of Arg, Trp, and Tyr in serum were significantly increased by the addition of AAAs. This suggests that LPS-induced immunity activated the mTOR pathway and increased the Arg requirements in animals. SLC38A2 participates in the regulation of AA availability (46) and also as an AA sensor upstream of mTOR (47). SLC38A2 knockdown in rat myocytes and leads to a drop in intracellular concentrations of both Gln and Leu (48). Gln has been demonstrated to be a rate-limiting nutrient for mTOR activation (49). The content of Gln in the jejunum mucosa was significantly decreased by the LPS stimulation. Compared to the basal diet group, the relative expression of SLC7A11 was significantly increased in the LPS-challenged group, especially in the jejunal mucosa. It promoted Cys uptake and Glu biosynthesis, resulting in protection from oxidative stress (50). The Cys levels were markedly increased both in jejunal mucosa and serum. SLC7A11 imports extracellular Cys with intracellular Glu release at a ratio of 1:1 (51). According to reports, SLC6A19 as a major transporter for neutral AAs is the main agent of branched-chain AAs and Met absorption in the intestinal tract (52). However, the AAA diet significantly reduced its expression in the jejunum, but had no significant effect on the content of three branched-chain AAs and Met in the jejunal mucosa. AAA diet up-regulated significantly the expression level of SLC16A10. This proves the roles of SLC16A10 in mediating facilitated diffusion of AAAs across membranes and maintaining homeostasis by balancing AAAs concentrations between plasma and liver cells (53). SLC16A10 realizes the regulation of neutral AAs through the recovery of aromatic substrates (54).

In conclusion, the present results showed that the inflammation induced by LPS altered the AAs metabolism including the AAs profiles in serum and intestinal mucosa, as well as apparent ileal digestibility of AAs, etc. However, dietary supplementation with AAAs showed to improve the AAs sensing and utilization, which may meet the high demands for specific AAs in response to inflammation and immune response and then exert the anti-inflammatory effects. These findings may provide guidelines for the use of AAAs in animal and human nutrition.

## Data Availability Statement

The original contributions presented in the study are included in the article/supplementary material, further inquiries can be directed to the corresponding author/s.

## Ethics Statement

The animal study was reviewed and approved by the Institutional Animal Care and Use Committee of the Institute of Subtropical Agriculture, Chinese Academy of Sciences (2013020).

## Author Contributions

QDu: writing—original draft. BT: supervision, project administration, and funding acquisition. JW: writing—review and editing. BH: data curation and supervision. JL, MK, KH, QDe, and YY: supervision. All authors contributed to the article and approved the submitted version.

## Funding

This work was supported by the National Natural Science Foundation of China (32072745), Earmarked Fund for China Agriculture Research System (CARS-35), and Innovation Province Project (2019RS3021).

## Conflict of Interest

The authors declare that the research was conducted in the absence of any commercial or financial relationships that could be construed as a potential conflict of interest.

## Publisher's Note

All claims expressed in this article are solely those of the authors and do not necessarily represent those of their affiliated organizations, or those of the publisher, the editors and the reviewers. Any product that may be evaluated in this article, or claim that may be made by its manufacturer, is not guaranteed or endorsed by the publisher.

## Abbreviations

AA: amino acid
AAA: aromatic amino acid
AID: apparent ileal digestibility
Ala: alanine
ALB: albumin
ALP: alkaline phosphatase
ALT: alanine transaminase
Arg: arginine
Asn: asparagine
Asp: aspartic acid
AST: aspartate aminotransferase
BUN: blood urea nitrogen
CaSR: calcium-sensing receptor
CCK: cholecystokinin
Cys: cysteine
EAA: essential amino acid
Gln: glutamine
Glu: glutamic acid
GLU: glucose
Gly: glycine
His: histidine
Ile: isoleucine
LDH: lactic dehydrogenase
Leu: leucine
LPS: lipopolysaccharide
Lys: lysine
Met: methionine
mTOR: mammalian target of rapamycin
NEAA: non-essential amino acid
Phe: phenylalanine
Pro: proline
Ser: serine
SLC: solute carrier
Thr: threonine
Tp: total protein
Trp: tryptophan
Tyr: tyrosine
Val: valine.
