# Corrigendum: The amino acids sensing and utilization in response to dietary aromatic amino acid supplementation in LPS-induced inflammation piglet model

**DOI:** 10.3389/fnut.2023.1234022

**Published:** 2023-06-14

**Authors:** Qing Duanmu, Bie Tan, Jing Wang, Bo Huang, Jianjun Li, Meng Kang, Ke Huang, Qiuchun Deng, Yulong Yin

**Affiliations:** ^1^College of Animal Science and Technology, Hunan Agricultural University, Changsha, China; ^2^Laboratory of Animal Nutritional Physiology and Metabolic Process, Key Laboratory of Agro-Ecological Processes in Subtropical Region, National Engineering Laboratory for Pollution Control and Waste Utilization in Livestock and Poultry Production, Institute of Subtropical Agriculture, Chinese Academy of Sciences, Changsha, China

**Keywords:** aromatic amino acid, amino acids sensing, transporters, sensors, piglets

In the published article, there was an error regarding the affiliations for Bie Tan. As well as having affiliation 2, they should also be affiliated to College of Animal Science and Technology, Hunan Agricultural University, Changsha, China.

The authors apologize for this error and state that this does not change the scientific conclusions of the article in any way. The original article has been updated.

